# War and pandemic do not jeopardize Germans’ willingness to support climate measures

**DOI:** 10.1038/s43247-023-00755-z

**Published:** 2023-04-03

**Authors:** Adrian Rinscheid, Sebastian Koos

**Affiliations:** 1 Radboud University Nijmegen, Environmental Governance and Politics Group, Nijmegen, The Netherlands; 2 University of Konstanz, Cluster of Excellence “The Politics of Inequality”, Konstanz, Germany

**Keywords:** Environmental studies, Climate-change mitigation, Psychology and behaviour

## Abstract

How do the impacts of acute crises influence citizens’ willingness to support different types of climate measures? An acute crisis can be understood either as an impediment or as an opportunity for climate change mitigation. In the first perspective, crisis impacts would create negative spill-overs and dampen citizens’ willingness to support climate action, while in the second perspective, the opposite would occur. Based on a survey experiment fielded in Germany in 2022 (*n* = 5438), we find that the economic implications of the Russo-Ukrainian War do not decrease behavioral willingness, while restrictions of civil liberties to combat the COVID-19 pandemic lead to higher climate support, underpinning the crisis-as-opportunity perspective. Willingness to support climate measures is strongest among (1) those most concerned about climate change, and (2) those who trust the government. We conclude that individuals do not wish climate change mitigation to be deprioritized on the back of other crises.

## Abstract (web summary)

The economic implications of the Russo-Ukrainian War and the impact of lockdown restrictions during the COVID-19 pandemic do not negatively affect public support for climate measures and suggest a crisis-as-opportunity perspective, according to a 2022 survey experiment with 5438 German residents.

## Introduction

Confronting the climate crisis requires governments around the world to take resolute and rapid action^1^. Despite the prospect of long-term collective gains from mitigating climate change, some of the required measures (which we call type 1) impose short-term costs on individuals, such as carbon taxes. Others (we call them type 2), like bans on combustion engine cars, are rather characterized by an encroachment of “individual freedom”—for example, the freedom to drive a diesel-fueled sports utility vehicle as far and as often as one desires^2^. While much attention has been devoted to type 1 measures, type 2 measures are no less important. An encompassing and effective approach to mitigating climate change very likely needs to consider both types of policies^1^. Crucially, in democracies, political measures of both types require public support. Otherwise, it is unlikely for them to be enacted, or they will even provoke public backlash^3^.

Climate change, according to the United Nations, is “the defining crisis of our time”^4^. At the same time, some say that humanity has even entered an age of “perpetual crisis”^5^ characterized by multiple and sometimes overlapping crises. Generally, crises are understood as major shocks affecting the livelihood of many people, such as natural disasters, wars, terrorist attacks, pandemics, economic shocks, or environmental catastrophes^6^. Europe has been particularly affected by two acute crises recently, the COVID-19 pandemic and the Russian invasion of Ukraine. Climate change is different from these acute crises, as it is not a sudden but a gradually emerging, long-term threat. The COVID-19 pandemic and the Russo-Ukrainian War, on the other hand, occurred unexpectedly for most people and rapidly generated impacts that affected societies as a whole.

While a vast literature has addressed the determinants of public support for climate policies^7–9^, studies rarely account for the importance of immediate crises and their potential impact on climate policy preferences^10^. Assuming that societies will be affected by crises other than climate change as we go, it is important to understand how such crises influence public views on climate change mitigation. To explore this broader question, we examine how the impacts of the Russo-Ukrainian War and the COVID-19 pandemic affect the public’s willingness to support climate action. We do so by investigating willingness to support type 1 and type 2 measures in Germany, the biggest economy and emitter of greenhouse gases in Europe. Like many other European countries, Germany has recently been strongly affected by the war, the pandemic, and climate change. We approach our research question by linking theories of crisis and the climate-related literature on public perceptions. Theoretically, we contrast two perspectives that suggest crises will either undermine (“crisis-as-stress”) or increase (“crisis-as-opportunity”) citizens’ willingness to support climate action. We contend that it ultimately depends on pre-existing beliefs, such as climate concern and trust in government, whether individuals reduce or increase their willingness to support climate measures.

Within the first perspective, societal crises are often described as periods of uncertainty and “collective stress”^11^. According to this dominant view, crises cause adverse psychological changes in those affected, such as increasing worries about how to cope with the emerging crisis^12^. This crisis-as-stress perspective shares similarities with the “finite pool of worry” hypothesis, which predicts that concern about climate change dwindles as other concerns gain in importance^13,14^. Accordingly, an acute crisis can be expected to undermine support for policies addressing the climate crisis. Along these lines, previous research has attempted to identify the extent to which economic downturns lower public concern for environmental issues including climate change^15,16^. Often proceeding from a classical rational actor perspective, the presumption is that individuals’ economic deprivation by a crisis reduces their willingness to contribute to public goods, along with increasing incentives to free-ride^17^. However, crises do not only encompass economic costs but might also limit people’s available time, well-being, or civil liberties. Some crises, for instance, have been accompanied by restrictions to civil liberties, such as the terrorist attacks on September 11, 2001, or the recent public health crisis surrounding the COVID-19 pandemic. When it comes to climate policy, research shows that perceived infringement on personal freedom tends to reduce people’s support^18^.

Yet, a second perspective emphasizes that crises can be opportunities for change. Rooted in the ancient Greek meaning, dating back to Plato, crisis was originally understood as a turning point, choice, or decision^19^. Indeed, prominent theories in economics^20^ and behavioral science^21^ maintain that crises can offer new problem frames and ideas, thereby providing opportunities for behavioral changes. Such changes may be driven by societal agents who point towards common roots, patterns, or implications of crises to suggest particular solutions. As research from public administration^22,23^ shows, decision-makers who perceive crises as an opportunity to enact changes will indeed try to propagate their interpretation among broader constituencies. Along these lines, several German policy-makers depicted the Russo-Ukrainian War as a catalyst for accelerating the transition of the economy away from fossil fuels. Another mechanism that could explain how perceptions of acute crises may positively influence willingness to mitigate climate change relies on the generalization of affect. According to this theory, concerns induced by an acute threat may spill over to other threats^24^, for instance due to the (mis)attribution of one threat to others, or because concern about one threat is transferred to another threat via associative networks^25,26^. Worry about the emergence of the COVID-19 health threat, for example, might also generalize to climate change. Insofar as increased climate concern predicts increased support for climate change mitigation^27^, this mechanism may lead us to expect that acute crises can provide an opportunity for stronger citizen commitment to climate change mitigation.

Here, we are especially interested in assessing whether different crises and their implications for individuals affect their willingness to support type 1 and type 2 climate measures in distinct ways. The Russo-Ukrainian War has far-reaching economic consequences for many countries. In Germany, as a consequence of the Russian invasion of the Ukraine, prices for gasoline and petroleum gas rose massively (+25% from January to March 2022, see ref. ^28^). While uncertainty about the economic effects of the war was extremely high during the first few months after the war onset (including the period when this study was fielded), prices for fuels were curbed considerably by strong government intervention later on. COVID-19 had economic implications as well, but in Germany was primarily associated with restrictions of personal freedom. While only 15% of Germans reported to be afraid of economic loss in 2020, restrictions of civil liberties were seen as a threat by one in four Germans^29^. The fact that economic worries remained limited during the pandemic is due to strong economic and social policy interventions in line with the institutional configuration in Germany, which largely follows the model of coordinated market economies and thereby differs from the situation in liberal market economies like the US^30^. We leverage this asymmetry for our study. Because Germans mainly perceived the Russo-Ukrainian War in terms of its immediate economic repercussions (apart from the obvious security threats), we used it to test how the salience of economic threats affect willingness to support type 1 policies. Conversely, the restrictions to civil liberties were the most salient issue associated with the COVID-19 pandemic in Germany. Therefore, these impacts of the crisis may have an influence on citizens’ willingness to support type 2 climate policies.

The impact of crises on willingness to support climate action might also depend on preexisting beliefs about climate change and individuals’ trust in their government to address crises effectively. In terms of climate change beliefs, prior research has particularly underscored the role of concern about climate change^27,31–33^. Accordingly, individuals who are not concerned about climate change are unlikely to support mitigation measures. However, it is less clear whether and how concern about climate change shapes citizens’ behavioral willingness under conditions of an immediate crisis. In line with the crisis-as-stress perspective, unconcerned individuals may even reduce their (already weak) support for climate action in an immediate crisis situation, as they focus on attenuating the direct threat rather than a threat perceived as distant in time and place^34,35^. For individuals that are already concerned about climate change, generalization of affect might play a particularly important role and increase willingness to support climate action even more in an acute crisis situation. For instance, concern about rising fuel prices may be linked with worries about how to satisfy mobility and energy needs in a carbon-constrained world, raising people’s willingness to make an investment into climate change mitigation. Hence, while we expect high climate concern to be linked with high climate policy support in general, support levels should be even higher among individuals confronted with an immediate crisis situation.

Research has documented that people’s willingness to support climate policies is positively related with their trust in government^8,36,37^. We expect that individuals who generally do not trust the government will be unlikely to change their perspective in times of an immediate crisis. Rather, a crisis may undermine a government’s perceived trustworthiness even further^38^. Hence, individuals with low trust in government who are exposed to immediate crises can be expected to further reduce their support for climate policies, in particular if these are perceived to entail additional costs^39^. Individuals with high trust in government, on the other hand, tend to view governmental crisis management in a more positive light in the first place. In a situation of immediate crisis, high trust in government could further increase the willingness to support climate action. In particular, such a trust spill-over effect may be expected if individuals’ trust in government is fueled by a positive assessment of recent governmental crisis management.

To investigate these theoretical expectations empirically, we designed a survey experiment in which respondents were randomly assigned to different crisis scenarios before they eventually indicated their willingness to support type 1 and type 2 climate measures. Our analysis suggests that neither the economic implications of the Russo-Ukrainian War nor the restriction of civil liberties to combat COVID-19 decrease individuals’ willingness to support climate action in Germany. Quite the contrary, COVID-19 mitigation policies even strengthen support for type 2 climate policies. This effect is particularly pronounced among respondents most concerned about climate change and individuals who trust the government.

## Results

### Study Design

In spring (April 12 to 25) 2022, we conducted a survey experiment. Participants (*n* = 5,438) were randomly assigned to one of four conditions. In each condition, participants were asked to imagine a particular situation to unfold in autumn. Participants assigned to the “War” condition were asked to imagine that the Russo-Ukrainian War intensifies, leading to a complete stop of Russian raw material deliveries to Germany and a drastic price increase for gas, electricity, food, and other goods. Individuals in the “COVID-19” condition were asked to imagine that a new mutation causes a severely worsened pandemic situation, leading to the adoption of policy measures that restrict personal freedoms in all areas of life. Importantly, we linked each crisis explicitly to its respective decisive consequence—rising prices (“War”) and freedom restrictions (“COVID-19”). Participants in the “Two crises” condition were asked to imagine both crisis scenarios to happen in autumn, with the crises stimuli being shown in random order across individuals. In the “No crisis” condition, the war was depicted as having ended, and the pandemic as mostly contained. Thus, in this condition, neither economic hardship nor freedom-related restrictions were relevant. Next, participants indicated the extent to which they were (a) personally willing to bear higher financial costs to mitigate climate change, and (b) personally willing to give up personal freedoms to mitigate climate change, resembling support for both type 1 and type 2 measures. Both dependent variables were measured on a seven-point scale from “very low” to “very high”.

### Findings

First, we briefly discuss the distribution of the dependent variables across the entire sample. For outcome (a), the distribution is somewhat right-skewed (mean = 3.154; SD = 1.832; skewness = 0.264), with 30.1% of respondents indicating a “very low” willingness to bear higher financial costs, and only 3.7% indicating a “very high” willingness (see Supplementary Fig. A). For outcome (b), the pattern is different. The distribution is slightly left-skewed (mean = 3.924; SD = 1.771; skewness = −0.187), with a much lower share of respondents indicating a “very low” willingness to give up personal freedoms (14.9%; see Supplementary Fig. B). Hence, our data suggest that in the context of climate change mitigation, Germans are generally more inclined to give up personal freedoms than to make an economic sacrifice. While we are not aware of another study measuring behavioral willingness to take a pass on personal freedoms in a German sample, we can compare the results of the costs outcome with an item used in the “Social Sustainability Barometer” in 2019. According to this representative survey (*n* = 6163), 40% of Germans were not willing to shoulder higher energy costs before COVID-19 and the Russian invasion of Ukraine^40^. This roughly corresponds to the share of respondents in our sample (42.1%) who had a very low or low willingness to bear higher financial costs (corresponding to 1 and 2 on the seven-point scale). Notwithstanding that the cited study does not lend itself to a straightforward comparison with ours, these numbers provide an indication that there has not been a sizeable drop in citizens’ willingness to bear higher costs for climate change mitigation after the onset of the crises.

**Fig. 1:**
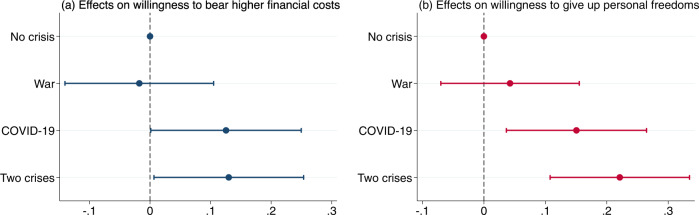
Average treatment effects on willingness to support climate change mitigation measures. **a** Effects on willingness to bear higher financial costs (type 1). **b** Effects on willingness to give up personal freedoms (type 2). The estimates are based on linear models controlling for demographics (age, education, gender), car usage, political orientation, climate change perceptions, and trust in science and government. Dots without bars indicate the reference category. Bars indicate 95% confidence intervals. We applied listwise deletion to deal with missing data. The results rely on **a** *n* = 4542 and **b** *n* = 4541 respondents. Full results are shown in Supplementary Table 1.

Next, we turn to our experimental results. Figure 1 shows the estimated effects of the experimental conditions on the willingness to support climate change mitigation. Compared with the “No crisis” baseline, the “War” condition did not significantly influence participants’ willingness to support type 1 (economic costs) or type 2 (freedom restrictions) measures. Yet, for both the “COVID-19” and the “Two crises” conditions, we obtained statistically significant positive effects. Hence, participants in these experimental conditions increased their average support level for both types of measures. With respect to type 1, the effects were almost the same for the “COVID-19” condition (*b* = 0.126 [0.001, 0.250], *t* = 1.98, *P* = 0.048) and the “Two crises” condition (*b* = 0.130 [0.006, 0.254], *t* = 2.06, *P* = 0.039). When assessing effects on type 2, the “COVID-19” condition (*b* = 0.151 [0.037, 0.265], *t* = 2.59, *P* = 0.010) was slightly less effective than the “Two crises” condition (*b* = 0.221 [0.108, 0.335], *t* = 3.82, *P* = 0.000).

**Fig. 2:**
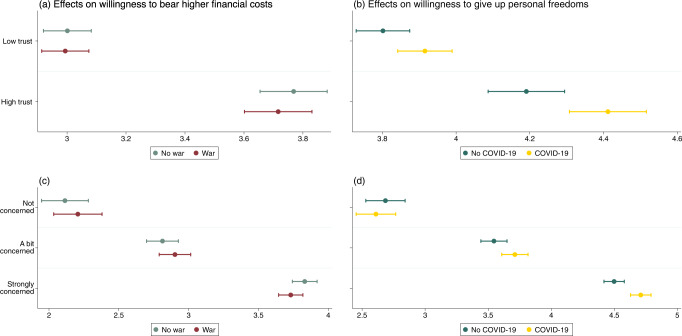
Heterogenous treatment effects on type 1 and type 2 measures. Heterogenous effects are illustrated based on adjusted predictions with 95% confidence intervals. Relies on linear regressions with interactions of pooled treatments with (**a**, **b**) trust in government and (**c**, **d**) concern about climate change. Results are shown in Supplementary Tables 3 and 4. Results based on non-pooled treatments lead to substantively similar conclusions.

As discussed earlier, we suspected that the effects could differ by trust in government and concern about climate change. Therefore, first, we focused on linear contrasts for pooled conditions highlighting economic hardship (“War” and “Two crises”) against the other conditions. As seen in Fig. 2a, trust in government had a sizeable main effect on support for type 1 measures, but did not moderate the relationship between war-induced economic hardship and support. The pattern was similar for climate concern (Fig. 2c). Second, we assessed the contrast between pooled conditions highlighting restrictions of personal freedom (“COVID-19” and “Two crises”) against the other conditions, with a view to understand support for type 2 measures. As seen in Fig. 2b, trust in government not only had a strong main effect on support, but, for individuals with high trust in government, also moderated the effect of COVID-19-related restrictions on the willingness to give up personal freedom. A similar pattern could be identified for people strongly concerned about climate change (Fig. 2d).

## Discussion

Modern societies frequently experience multifold crises, potentially shifting away much needed public support for climate change mitigation. Against this background, our study provides three important insights. First, acute crises do not need to undermine willingness to support climate change mitigation, neither for economically costly measures (type 1), nor for measures that restrict individual freedom (type 2). Similar to recent research^10,25,26,41,42^, we hence do not find evidence for the “finite pool of worry” hypothesis. Rather, in line with the idea of a “finite pool of attention”^25^, acute crises may shift people’s attention towards immediate threats, but do on average not reduce their willingness to support climate change mitigation. At the broadest level, this suggests that policymakers should not be tempted to cut back on climate measures when faced with other crises.

Second, crises predominantly characterized by economic fallout (in our context the Russian war) do not need to negatively affect citizens’ willingness to support even costly type 1 climate measures. While this finding is at odds with standard economic assumptions and older empirical studies (e.g.^16,43^,), it is consistent with more recent research studying the impact of economic recessions on support for climate policy, which relies on more sophisticated measures of public opinion and stronger empirical identification strategies^44,45^. Consequently, as Mildenberger and Leiserowitz^45^ comment on their null results, “future policymakers may have broader latitude to address the climate threat across a range of economic circumstances”.

However, the crisis-as-opportunity perspective also has its limits in the context of the Russian war, the economic repercussions of which do not seem to be broadly perceived as an opportunity for climate change mitigation among Germans. This finding is in line with the extreme uncertainty provoked by the war, which was pervading Germany especially in the first few months after the invasion during spring 2022. While there was an elite discourse portraying the war as an opportunity to speed up the decarbonization of Germany’s energy sector early on (e.g.^46,47^,), measures to alleviate the immediate economic consequences of the war were only taken in May 2022 (after our survey was fielded).

Third, our findings on the impact of a crisis predominantly characterized by restrictions of civil liberties (COVID-19) lend some support to the crisis-as-opportunity perspective. Thus, under specific conditions and in line with Bergquist et al.’s recent study of climate policy preferences among US and Canadian publics^10^, an acute crisis seemingly unrelated to climate change can contribute to an increased support for long-term climate change mitigation. This may encourage policymakers to harness the synergies that can arise from strategically addressing multiple crises^48,49^. It also corroborates recent findings that the public is more willing to accept type 2 measures than many believe, including bans on unsustainable behaviors and other restrictive measures^50,51^.

Importantly, based on the example of COVID-19, our study goes beyond previous research in that it identifies the scope conditions under which the crisis-as-opportunity perspective is likely to hold. First, in line with existing research^32,52^, the role of climate concern is decisive. It is primarily those citizens that are already concerned about climate change who further increase their willingness to support climate measures in the presence of restrictions to freedom. This appears to be consistent with the mechanism of “affect generalization”, according to which spill-over effects could further increase these people’s concern about climate change, which in turn fuels behavioral willingness. In societies with little concern about climate change, this mechanism is unlikely to substantially alter behavioral willingness to support climate action. In Germany, however, the acute threat from COVID-19 met with the favorable condition of relatively high levels of climate concern (see also ref. ^53^).

Second, trust in government, which tends to be associated with a belief in the capacity of the state to effectively alleviate crises, also plays an important role in explaining willingness to accept freedom-restrictive climate measures. The underlying process is likely driven by spill-over effects as well, the nature of which, however, is primarily cognitive rather than affective. Among individuals that trust in the capacity of the state, the pandemic crisis management, which for the case of Germany has variously been evaluated as effective in comparison with other countries^54^, might have become an example leading the way to support climate policies that entail some restrictions for individuals. This may have been fueled by the often discussed commonalities between COVID-19 and the climate crisis, such as the preparedness paradox, which means that mitigation is less costly than adaptation, and the property that crossing certain thresholds can make the entire system uncontrollable^55^. Our conclusions on the role of trust in government are consistent with recent findings from the UK^56^ and Spain^57^ that highlight how trust in government can increase people’s willingness to support climate action during pandemic conditions. Our findings also suggest that in countries with very low trust in government, acute crises are unlikely to increase willingness to support climate change mitigation.

This is not to say that there are not important differences between climate change and other crises. For instance, while the time scales at which climate change unfolds involve a rather gradual but accelerating development over time, the COVID-19 pandemic was characterized by a rapid onset. Moreover, the link between cause and effects is relatively straightforward for COVID-19, with the consequences playing out quickly and at a personal level for many people, while cause-effect relationships are more complex and consequences less clearly attributable in the context of climate change^56,58,59^. Arguably, the Russian war is yet quite different from both COVID-19 and climate change in many respects, since its predictability and the “possibilities to influence” the crisis^6^ are much more limited.

Our interpretations of the study findings must be seen in light of some choices we made while designing the experiment. First, a central motivation of the study was to provide experimental evidence to enhance our understanding of ongoing societal phenomena. Hence, study participants were already familiar with the broader context of the pandemic and the war before entering the experiment, which differs from a typical lab experimental context. This particular setting may have affected the strength of the experimental manipulations compared to a situation in which there was no real-world pandemic and war, although there is no clear benchmark against which to judge how strong and in what direction such an effect would be. We deem it plausible to assume that “true” lab conditions might have produced even stronger effects, as individuals assigned to the “No crisis”-condition in our experiment are likely to have a higher baseline perception of the severity of the ongoing crises compared to the counterfactual (see^60^). This, in turn, would even strengthen the conclusions we draw from our study.

Second, and relatedly, we deliberately refrained from including a “true” control condition in which participants would not have received information about either crisis. Due to the high salience of both crises at the time of the fielding period, such a “neutral” control would in fact have lessened our control over the experiment and would, instead, have posed intricate challenges in terms of interpretation^61^. For instance, when comparing a neutral condition to the “no crisis” condition, effects in any direction could be driven by priming effects as much as by uncontrolled baseline perceptions of the crises among those assigned to the neutral condition. By assigning participants to clearly specified conditions, we neutralize the different baselines from which participants start. This enables us to interpret causal effects in a straightforward way based on the clearly specified crises scenarios.

What is more, the fact that our experiment was conducted in times of unfolding crises may strengthen the external validity of our findings^62^. Compared to a more artificial lab situation in which participants may have difficulties to imagine an ongoing pandemic or war (and would probably not find such scenarios overly plausible), the contextual conditions make the scenarios more salient. We are therefore confident that the findings have relevant implications for the real world^63^. However, as noted by Kinder^64^, “experimental results can always be questioned on their generalizability”. In our case, important limits to generalizability lie in the timing of the experimental intervention and the specific country context.

The results of our study are clearly limited to Germany. Yet, our study mirrors recent findings regarding the impact of the Russo-Ukrainian War on policy support among Swiss citizens^65^ and the effects of COVID-19 on climate concern among UK residents^41^. Keeping in mind the limitations of our study, we conclude that individuals do not wish climate change mitigation to be deprioritized on the back of other crises. Whether our findings also apply to other societies remains up to empirical explorations.

## Methods

The survey experiment was targeted at German residents aged 18 and older and representative in terms of gender, age, and education (according to the German Mikrozensus, https://www.gesis.org/en/gml/microcensus). Regarding the regional distribution, selection probabilities were higher for East Germans. We used regression weights to correct for this. Since the results are substantively the same, we report results from regression analyses with unweighted data in the paper.

Study participants were drawn from the online access panel operated by Bilendi. The panel is actively managed by a professional team and subject to permanent quality control in a scoring and monitoring process. The following exclusion criteria were implemented: dropout during the survey, nonsense responses to open questions, speeders, and straight lining. We have obtained informed consent from all study participants and ensured compliance with all relevant ethical regulations and guidelines for study procedures set forth by the Institutional Review Board of the University of Konstanz (reference no. 20KN09-006).

The precise wording of experimental interventions and outcome variables can be found in Supplementary Notes 1 and 2. We used linear regressions to estimate average treatment effects (Fig. 1). The estimates are based on linear models controlling for demographics (age, education, gender), car usage, political orientation, climate change perceptions, and trust in science and government (see Supplementary Table 2).

For the analysis of heterogeneous treatment effects (Fig. 2), we conducted separate linear contrast models for (a) pooled conditions highlighting economic hardship (“War” and “Two crises”) against the other conditions, and (b) pooled conditions highlighting restrictions of personal freedom (“COVID-19” and “Two crises”) against the other conditions. For the interactions with trust in government, we created a binary trust measure. Accordingly, “high trust” (31.8% of respondents) includes all study participants indicating (rather) high trust in government (values 5, 6, and 7 on the seven-point scale), and “low trust” (68.2%) includes all other respondents (values 1 to 4). For the interactions with concern about climate change, we generated a three-point scale based on the original five-point measure. Accordingly, “not concerned” (16.2% of respondents) includes participants indicating they were “not concerned at all” or “not very concerned” about climate change, “a bit concerned” (31.1%) corresponds to the mid-point on the original scale, and “strongly concerned” (52.7%) includes participants that indicated they were “very much” or “extremely” concerned (see Supplementary Table 2).

### Reporting summary

Further information on research design is available in the Nature Portfolio Reporting Summary linked to this article.

## Supplementary information

Supplementary Information

Reporting Summary

## Data Availability

Replication data for the study are available in the Harvard Dataverse with the identifier 10.7910/DVN/3VPRGG^66^.

## Code availability

Replication code for the study is available in the Harvard Dataverse with the identifier 10.7910/DVN/3VPRGG^66^.
