# Supplementary material for: War and pandemic do not jeopardize Germans’ willingness to support climate measures
Source: Commun Earth Environ. 2023 Apr 3;4(1):101. doi: 10.1038/s43247-023-00755-z (PMC10068711; doi:10.1038/s43247-023-00755-z)
Supplement: Supplementary file 1 — Supplementary Information [file 43247_2023_755_MOESM1_ESM.pdf]

Supplementary Information to:

War and pandemic do not jeopardize Germans' willingness to support climate measures

by Adrian Rinscheid & Sebastian Koos

Supplementary Note 1. Wording and distribution of outcome variables.

Outcome variables were shown in random order across participants.

Type 1 measures: Willingness to bear higher financial costs

"In such a situation, to what extent are you personally willing to bear higher financial costs to mitigate climate change?"

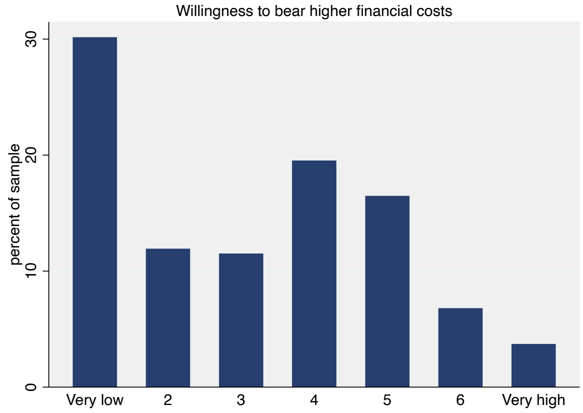

Supplementary Figure A. Distribution of cost outcome, full sample (n = 5,381, mean = 3.154, SD = 1.832). Assessed on a 7-point scale with labeled end points as shown in the figure.

Type 2 measures: Willingness to give up personal freedoms

"In such a situation, to what extent are you personally willing to give up personal freedoms to mitigate climate change?"

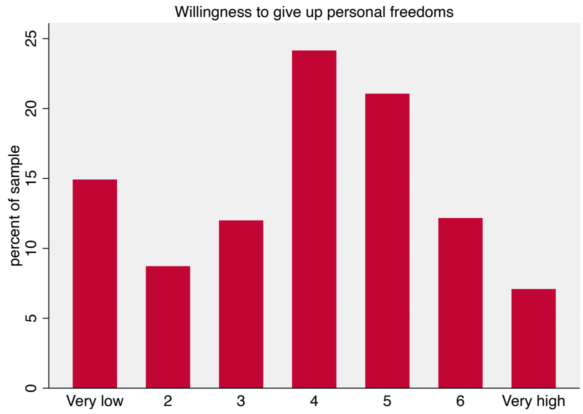

Supplementary Figure B. Distribution of freedom outcome, full sample (n = 5,374, mean = 3.924, SD = 1.771). Assessed on a 7-point scale with labeled end points as shown in the figure.

Supplementary Note 2. Experimental Conditions.

Interventions were shown in German in the experiment; here we display the English translations. Study participants saw the text in italics. The order of the first and second part of interventions (separated by line break below) was randomly displayed across participants within a condition.

Currently, apart from climate change, different other crises dominate media reports in Germany. Please imagine the following situation in autumn:

War condition (n = 1,379)

> *The* ***war in Ukraine*** *is leading to a halt in Russian deliveries of raw materials to Germany. This makes* ***many goods such as gas, electricity, gasoline or food much more expensive*** *and economic growth is slowed.*
> *The* ***Corona pandemic*** *is over.* ***No more restrictions on personal freedoms*** *are necessary to fight the pandemic.*

COVID-19 condition (n = 1,336)

> *The* ***Corona pandemic*** *has* ***worsened again*** *due to a mutation that leads to* ***many infections and deaths****. As a result, far-reaching measures are adopted that* ***restrict personal freedoms*** *in all areas of life (work, education, leisure).*
> *The* ***war in Ukraine has ended****. The conflict* ***no longer has far-reaching economic consequences*** *for the citizens in Germany.*

Two crises condition (n = 1,346)

> *The* ***war in Ukraine*** *is leading to a halt in Russian deliveries of raw materials to Germany. This makes* ***many goods such as gas, electricity, gasoline or food much more expensive*** *and economic growth is slowed.*
> *At the same time, the* ***Corona pandemic*** *has* ***worsened again*** *due to a mutation that leads to* ***many infections and deaths****. As a result, far-reaching measures are adopted that* ***restrict personal freedoms*** *in all areas of life (work, education, leisure).*

No crisis condition (n = 1,377)

> *The* ***war in Ukraine has ended****. The conflict* ***no longer has far-reaching economic consequences*** *for the citizens in Germany.*
> *At the same time, the* ***Corona pandemic*** *is over.* ***No more restrictions on personal freedoms*** *are necessary to fight the pandemic.*

Supplementary Table 1. Regression results.
Willingness to support type 1 / type 2 measures regressed on experimental conditions and covariates (see Fig. 1a and 1b in the manuscript). Regression coefficients are unstandardized.

	Effect on willingness to bear higher financial cost (type 1 measures)	Effect on willingness to give up personal freedoms (type 2 measures)
No crisis (baseline)		
War	-0.0177 (0.0626)	0.0427 (0.0575)
COVID-19	0.1255* (0.0634)	0.1506** (0.0582)
Two crises	0.1300* (0.0630)	0.2212*** (0.0579)
Age	-0.0723*** (0.0177)	0.0137 (0.0162)
Gender (baseline male)	-0.2610*** (0.0470)	0.0853* (0.0431)
Education	0.1523*** (0.0305)	0.0013 (0.0280)
Pol. orientation (baseline left)	-0.0771** (0.0293)	-0.0739** (0.0269)
Climate concern	0.6015*** (0.0260)	0.7020*** (0.0238)
Perceived scientific consensus	0.0182 (0.0229)	0.0110 (0.0210)
Certainty about climate change	-0.0614 (0.0364)	0.0261 (0.0334)
Trust in government	0.3235*** (0.0166)	0.2116*** (0.0152)
Trust in science	0.0295 (0.0182)	0.1223*** (0.0167)
Car usage	-0.0770 (0.0457)	-0.1570*** (0.0420)
constant	0.2821 (0.1944)	0.2989 (0.1783)
N	4542	4541
R^2	0.343	0.400

Standard errors in parentheses
* $p < 0.05$, ** $p < 0.01$, *** $p < 0.001$

Supplementary Table 2. Control variables used in the analyses.

Age	1. 18-29 (13.02%)<br>2. 30-39 (15.39%)<br>3. 40-49 (17.21%)<br>4. 50-59 (26.52%)<br>5. 60+ (27.86%)<br>*No missing values*
Gender	0. Male (53.38%)<br>1. Female (46.52%)<br>2. Other (0.18%) – *excluded from analyses*<br>*No missing values*
Education	1. Low level of education (29.62%)<br>2. Medium level of education (34.68%)<br>3. High level of education (35.69%)<br>*No missing values*
Political orientation	1. Left (30.64%)<br>2. Center (30.03%)<br>3. Right (27.51%)<br>*No response / Don't know: 11.82%*
Climate concern	1. Not concerned at all (6.12%)<br>2. Not very concerned (9.64%)<br>3. A bit concerned (30.34%)<br>4. Very much concerned (35.49%)<br>5. Extremely concerned (15.89%)<br>*No response / Don't know: 2.52%*
Perceived scientific consensus	1. Up to 40% of scientists agree about man-made climate change (22.84%)<br>2. More than 40% and up to 60% of scientists agree (21.06%)<br>3. More than 60% and up to 80% of scientists agree (27.49%)<br>4. More than 80% of scientists agree (28.61%)<br>*No missing values*
Certainty about climate change	1. Climate is definitely not changing (2.61%)<br>2. Climate is probably not changing (5.74%)<br>3. Climate is probably changing (25.73%)<br>4. Climate is definitely changing (62.12%)<br>*No response / Don't know: 3.81%*
Trust in government	1. No trust at all (22.21%)<br>2. *Not labeled* (11.68%)<br>3. *Not labeled* (14.69%)<br>4. *Not labeled* (19.66%)<br>5. *Not labeled* (19.11%)<br>6. *Not labeled* (9.32%)<br>7. Very strong trust (3.05%)<br>*No response: 0.28%*

Trust in science	1. No trust at all (8.05%)<br>2. *Not labeled* (5.42%)<br>3. *Not labeled* (10.46%)<br>4. *Not labeled* (18.13%)<br>5. *Not labeled* (20.39%)<br>6. *Not labeled* (23.87%)<br>7. Very strong trust (13.24%)<br>*No response: 0.42%*
Car usage	1. Car usage up to once per week (43.97%)<br>2. Car usage more than once per week (55.68%)<br>*No response / Don't know: 0.35%*

Supplementary Table 3. Regression results with interaction effects, type 1.
Willingness to support type 1 measures regressed on experimental conditions and covariates, including interaction effects with trust in government and concern about climate change (see Fig. 2a and 2c in the manuscript). Regression coefficients are unstandardized.

	Effect on willingness to bear higher financial cost (type 1 measures)	
*No war*Low trust (baseline)*	-	
No war*High trust	0.773***(0.0741)	
War*Low trust	-0.00734(0.0564)	
War* High trust	0.717***(0.0742)	
*No war*Not concerned*		-
No war*A bit concerned		0.698***(0.101)
No war*Strongly concerned		1.717***(0.101)
War*Not concerned		0.0925(0.115)
War*A bit concerned		0.788***(0.101)
War*Strongly concerned		1.617***(0.101)
Age	-0.0923***(0.0181)	-0.0818***(0.0178)
Gender (baseline male)	-0.258***(0.0482)	-0.252***(0.0471)
Education	0.133***(0.0313)	0.140***(0.0306)
Pol. orientation (baseline left	-0.107***(0.0300)	-0.0836**(0.0294)
Climate concern	0.894***(0.0380)	
Perceived scientific consensus	0.0140(0.0234)	0.0260(0.0229)
Certainty about climate change	-0.0571(0.0375)	-0.0556(0.0367)
Car usage	-0.104*(0.0468)	-0.0874(0.0459)
Trust in government		0.324***(0.0166)
Trust in science	0.135***(0.0171)	0.0338(0.0183)
constant	0.902***(0.197)	1.327***(0.198)
N	4542	4542
R^2	0.309	0.338

Standard errors in parentheses
* $p < 0.05$, ** $p < 0.01$, *** $p < 0.001$

Supplementary Table 4. Regression results with interaction effects, type 2.
Willingness to support type 2 measures regressed on experimental conditions and covariates, including interaction effects with trust in government and concern about climate change (see Fig. 2b and 2d in the manuscript). Regression coefficients are unstandardized.

	Effect on willingness to give up personal freedoms (type 2 measures)	
*No COVID-19*Low trust (baseline)*	-	
No COVID-19*High trust	0.387***(0.0671)	
COVID-19*Low trust	0.114*(0.0513)	
COVID-19* High trust	0.609***(0.0673)	
*No COVID-19*Not concerned*		-
No COVID-19*A bit concerned		0.861***(0.0932)
No COVID-19*Strongly concerned		1.814***(0.0933)
COVID-19*Not concerned		-0.0755(0.105)
COVID-19*A bit concerned		1.026***(0.0934)
COVID-19*Strongly concerned		2.025***(0.0934)
Age	-0.00365(0.0165)	0.00324(0.0163)
Gender (baseline male)	0.0875*(0.0439)	0.0925*(0.0433)
Education	-0.0205(0.0285)	-0.0155(0.0281)
Pol. orientation (baseline left)	-0.0991***(0.0273)	-0.0843**(0.0270)
Climate concern	1.018***(0.0345)	
Perceived scientific consensus	0.0126(0.0213)	0.0208(0.0211)
Certainty about climate change	0.0299(0.0341)	0.0326(0.0337)
Car usage	-0.177***(0.0427)	-0.166***(0.0422)
Trust in government		0.211***(0.0153)
Trust in science	0.200***(0.0156)	0.127***(0.0168)
constant	0.763***(0.178)	1.567***(0.181)
N	4541	4541
R^2	0.379	0.395

Standard errors in parentheses
* $p < 0.05$, ** $p < 0.01$, *** $p < 0.001$
