# Supplementary material for: War and pandemic do not jeopardize Germans’ willingness to support climate measures
Source: Commun Earth Environ. 2023 Apr 3;4(1):101. doi: 10.1038/s43247-023-00755-z (PMC10068711; doi:10.1038/s43247-023-00755-z)
Supplement: Supplementary file 2 — Reporting Summary [file 43247_2023_755_MOESM2_ESM.pdf]

nature portfolio

Corresponding author(s): Adrian Rinscheid

Last updated by author(s): Feb 24, 2023

Reporting Summary

Nature Portfolio wishes to improve the reproducibility of the work that we publish. This form provides structure for consistency and transparency in reporting. For further information on Nature Portfolio policies, see our Editorial Policies and the Editorial Policy Checklist.

Statistics

For all statistical analyses, confirm that the following items are present in the figure legend, table legend, main text, or Methods section.

n/a	Confirmed	
☐	☒	The exact sample size (*n*) for each experimental group/condition, given as a discrete number and unit of measurement
☐	☒	A statement on whether measurements were taken from distinct samples or whether the same sample was measured repeatedly
☐	☒	The statistical test(s) used AND whether they are one- or two-sided<br>*Only common tests should be described solely by name; describe more complex techniques in the Methods section.*
☐	☒	A description of all covariates tested
☒	☐	A description of any assumptions or corrections, such as tests of normality and adjustment for multiple comparisons
☐	☒	A full description of the statistical parameters including central tendency (e.g. means) or other basic estimates (e.g. regression coefficient) AND variation (e.g. standard deviation) or associated estimates of uncertainty (e.g. confidence intervals)
☐	☒	For null hypothesis testing, the test statistic (e.g. *F*, *t*, *r*) with confidence intervals, effect sizes, degrees of freedom and *P* value noted<br>*Give P values as exact values whenever suitable.*
☒	☐	For Bayesian analysis, information on the choice of priors and Markov chain Monte Carlo settings
☒	☐	For hierarchical and complex designs, identification of the appropriate level for tests and full reporting of outcomes
☒	☐	Estimates of effect sizes (e.g. Cohen's *d*, Pearson's *r*), indicating how they were calculated

Our web collection on statistics for biologists contains articles on many of the points above.

Software and code

Policy information about availability of computer code

Data collection	Data collection was carried out by external data provider Bilendi
Data analysis	STATA15

For manuscripts utilizing custom algorithms or software that are central to the research but not yet described in published literature, software must be made available to editors and reviewers. We strongly encourage code deposition in a community repository (e.g. GitHub). See the Nature Portfolio guidelines for submitting code & software for further information.

Data

Policy information about availability of data

All manuscripts must include a data availability statement. This statement should provide the following information, where applicable:

- Accession codes, unique identifiers, or web links for publicly available datasets
- A description of any restrictions on data availability
- For clinical datasets or third party data, please ensure that the statement adheres to our policy

Replication data and code for the study are available in the Harvard Dataverse with the identifier https://doi.org/10.7910/DVN/3VPRGG

Human research participants

Policy information about studies involving human research participants and Sex and Gender in Research.

Reporting on sex and gender	We used self-reported gender by study participants. We did not expect gender differences in our analysis and our results support this expectation. n(female)=2,525; n(male)=2,903
Population characteristics	See section "Research sample" below.
Recruitment	In partnership with the online survey firm Bilendi panelists were invited to participate in a study on attitudes in the context of ongoing crises. Participation was voluntary.
Ethics oversight	This study was approved by the ethics committee of the University of Konstanz. All subjects provided informed consent.

Note that full information on the approval of the study protocol must also be provided in the manuscript.

Field-specific reporting

Please select the one below that is the best fit for your research. If you are not sure, read the appropriate sections before making your selection.

☐ Life sciences ☒ Behavioural & social sciences ☐ Ecological, evolutionary & environmental sciences

For a reference copy of the document with all sections, see nature.com/documents/nr-reporting-summary-flat.pdf

Behavioural & social sciences study design

All studies must disclose on these points even when the disclosure is negative.

Study description	The quantitative survey data were collected as part of a multi-module cross-sectional survey. The data for this study is (survey) experimental; we conducted a factorial survey experiment.
Research sample	Panelists of a commercial panel in Germany. Participants had to be at least 18 years old and reside in Germany. Panelists were selected according to their gender (2 groups), age (5 groups), education (3 groups) and region, distinguishing between east and west Germany (2 groups) to approximate marginal distributions of the Micro-census in 2018. We used a non-probability quota sample.
Sampling strategy	Participants were recruited from a commercial online access panel administered and remunerated by Bilendi. Membership and participation is voluntary and follows a double opt-in registration process. Participation is incentivized with tokens which can be exchanged for goods.
Data collection	The survey design and programming (including the randomized assignment to treatments) was implemented on our end, while Bilendi rewarded respondents with their token system.
Timing	April 12 to 25, 2022.
Data exclusions	The following exclusion criteria were defined before data collection: dropout during the survey, speeders, and straight lining.
Non-participation	N/A
Randomization	In the experiment, a randomly selected vignette from the vignette universe was assigned to each survey respondent.

Reporting for specific materials, systems and methods

We require information from authors about some types of materials, experimental systems and methods used in many studies. Here, indicate whether each material, system or method listed is relevant to your study. If you are not sure if a list item applies to your research, read the appropriate section before selecting a response.

Materials & experimental systems

n/a	Involved in the study
☒	☐ Antibodies
☒	☐ Eukaryotic cell lines
☒	☐ Palaeontology and archaeology
☒	☐ Animals and other organisms
☒	☐ Clinical data
☒	☐ Dual use research of concern

Methods

n/a	Involved in the study
☒	☐ ChIP-seq
☒	☐ Flow cytometry
☒	☐ MRI-based neuroimaging
